# A new genus of oonopid spiders from Myanmar (Araneae, Oonopidae)

**DOI:** 10.3897/zookeys.794.29156

**Published:** 2018-11-01

**Authors:** Yanfeng Tong, Haifeng Chen, Sijia Liu, Shuqiang Li

**Affiliations:** 1 Life Science College, Shenyang Normal University, Shenyang 110034, China Shenyang Normal University Shenyang China; 2 College of Life Science, Langfang Normal University, Langfang 065000, Hebei Province, China Langfang Normal University Langfang China; 3 Institute of Zoology, Chinese Academy of Sciences, Beijing 100101, China Institute of Zoology, Chinese Academy of Sciences Beijing China

**Keywords:** New species, Oonopinae, Southeast Asia, Taxonomy

## Abstract

A new genus, *Kachinia* Tong & Li, **gen. n.**, including two new species, *K.putao* Tong & Li, **sp. n.**, and *K.mahmolae* Tong & Li, **sp. n.**, is described from Myanmar. The new genus belonging to the subfamily Oonopinae Simon, 1890, currently is the only member of the *Brignolia*-*Opopaea* clade with leg spines.

## Introduction

The taxonomy of the family Oonopidae Simon, 1890 has a huge progressed in the last decade, more than 1,300 new species, and 50 new genera were described in the past ten years. This spider family currently includes 1,801 extant described species in 114 genera (WSC 2018), making it the 8^th^ most speciose spider family so far following the hyperdiverse spider groups such as jumping spiders (Salticidae Blackwall, 1841). The Oonopidae were traditionally divided into two informal groups, the “loricati”, harboring oonopids with heavily sclerotized bodies, and the “molles”, which includes the remaining, soft-bodied goblin spiders (Simon 1893). This fundamental dichotomy was later fixed as formal categories at subfamily level, Gamasomorphinae Petrunkevitch, 1923 for the “loricati” and Oonopinae Simon, 1890 for the “molles” (Petrunkevitch 1923). Platnick et al. (2012a) clarified the classification of the oonopids. Three subfamilies were recognized, i.e., Oonopinae, Orchestininae Chamberlin and Ivie, 1942, and Sulsulinae Platnick, 2012. The subfamily Oonopinae harbor the bulk of oonopid genera, including those groups formerly regarded as belonging to ‘‘Gamasomorphinae’’. However, the task of recognizing homologous instances of modification on the body sclerotization will play a central role in future efforts to detail the classification of the higher Oonopinae (Bonaldo et al. 2014).

The oonopid spiders of Myanmar have been poorly studied. Hitherto, only four extant species, *Gamasomorphabipeltis* Thorell, 1895, *G.inclusa* Thorell, 1887, *G.psyllodes* Thorell, 1897 and *G.sculptilis* Thorell, 1897, have been recorded from Myanmar (WSC 2018). The present paper expands the known oonopid diversity of Myanmar by adding a new genus and two new species.

## Materials and methods

The specimens were examined using a Leica M205C stereomicroscope. Details were studied under an Olympus BX51 compound microscope. Photos were made with a Canon EOS 550D zoom digital camera (18 megapixels) mounted on an Olympus BX51 compound microscope. Vulvae were cleared in lactic acid. Scanning electron microscope images (SEM) were taken under high vacuum with a Hitachi S-4800 after critical point drying and gold-palladium coating. All measurements were taken using an Olympus BX51 compound microscope and are in millimeters.

References to figures in the cited papers are listed in lowercase (figure or figs); figures from this paper are noted with an initial capital (Figure or Figs). The following abbreviations are used in the text and figures (Terminology partly follows Eichenberger and Kranz-Baltensperger 2011; Grismado et al. 2014):

**ALE** anterior lateral eyes;

**ap** apodemes;

**asr** anterior scutal ridge;

**blp** broom-like projection;

**boc** booklung cover;

**bpr** brush-like projection;

**clo** curved lobe;

**fil** filiform lobe;

**fls** feather-like setae;

**ibr** inner branch;

**ldi** deep incision of labium;

**lel** large, ear-shaped lobe;

**llp** leaf-like projection;

**lse** long setae;

**lst** line-like structure;

**obr** outer branch;

**pl** plate;

**PLE** posterior lateral eyes;

**PME** posterior median eyes;

**pr** posterior receptacle;

**psp** posterior spiracles;

**sar** sclerotized, recurved arches;

**sls** strong, long setae;

**tsc** T-shaped sclerite (= AS of Grismado et al. 2014);

**vp** ventral protuberance.

The type material is deposited in the Institute of Zoology, Chinese Academy of Sciences in Beijing (**IZCAS**).

## Taxonomy

### Kachinia

Taxon classificationAnimaliaAraneaeOonopidae

Tong & Li gen. n.

http://zoobank.org/3380B0EC-974B-483C-916D-44CBA425FBFC

#### Type species.

*Kachiniaputao* Tong & Li, sp. n.

#### Etymology.

The generic name is derived from the type locality, ‘Kachin’, and is feminine in gender.

#### Diagnosis.

*Kachinia* gen. n. resembles *Brignolia* Dumitrescu & Georgescu, 1983, the member of *Brignolia*-*Opopaea* clade (De Busschere et al. 2014), by the heavily sclerotized and darkened palps of males, and the endogyne bearing a T-shaped anterior sclerite and tube-like posterior receptacle, but can be easily distinguished by the presence of anterior leg spines in both sexes, the deeply incised labium and the branched endites in males and the absence of external features of endogyne (*B.parumpunctata* (Simon, 1893) with external modifications, see Platnick et al. 2011: figure 69). The new genus is also similar to *Ischnothyreus* Simon, 1893, but can be separated by the deeply incised labium, the branched endites, the unmodified chelicerae and the totally fused cymbium and bulb in males (*Ischnothyreuspeltifer* (Simon, 1892), and most species of this genus usually with a tooth-like projection on the anteromedian tip of the endites, with processes on the base of the cheliceral fang and cymbium fused with bulb but with clearly defined seam, see Kranz-Baltensperger 2011: figure 1C; Richard et al. 2016: figure 19C, D; Platnick et al. 2012b: figs 7–11, 34; Tong et al. 2018: figure 1e) and the T-shaped anterior sclerite (tsc, Figs 3J, 6J) and tube-like posterior receptacle (pr, Figs 3J, 6J) of the endogyne (*Ischnothyreus* has an elongated, highly curved sclerotized duct). The new genus is also similar to *Trilacuna* Tong & Li, 2007 because of the modifications to the male labium and endites, but can be distinguished by the heavily sclerotized palps, the egg-shaped patches behind the eyes in males, the smooth sides of the carapace (*Trilacunarastrum* Tong & Li, 2007 has granulates on the sides of carapace, see Tong and Li 2007: figure 1) and the large plate (pl, Figs 3I, 6I) in the endogyne (only *T.kropfi* Eichenberger, 2011 with a small semicircular plate, see Eichenberger and Kranz-Baltensperger 2011: figure 18B).

#### Description.

**Figure 1.**
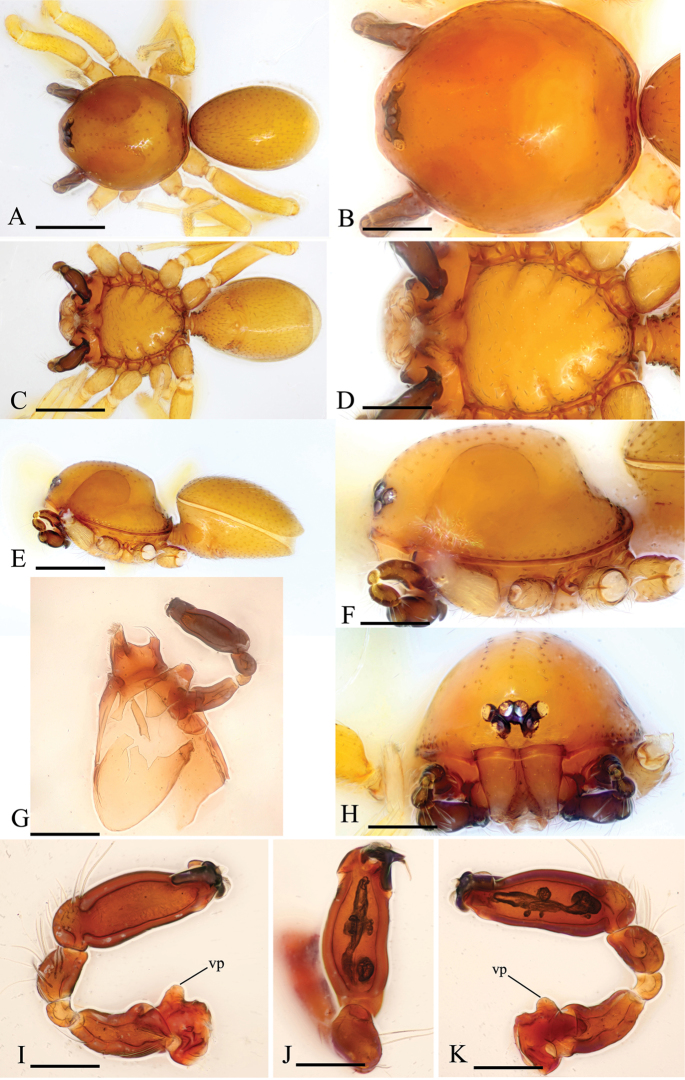
*Kachiniaputao* sp. n., holotype male. **A, C, E** habitus, dorsal, ventral and lateral views **B, D, F, H** prosoma, dorsal, ventral, lateral and anterior views **G** detached left endite and palp, ventral view **I, J, K** left palp, prolateral, dorsal and retrolateral views. Abbreviation: vp = ventral protuberance. Scale bars: 0.4 mm (**A, C, E**); 0.2 mm (**B, D, F, G, H**); 0.1 mm (**I, J, K**).

**Figure 2.**
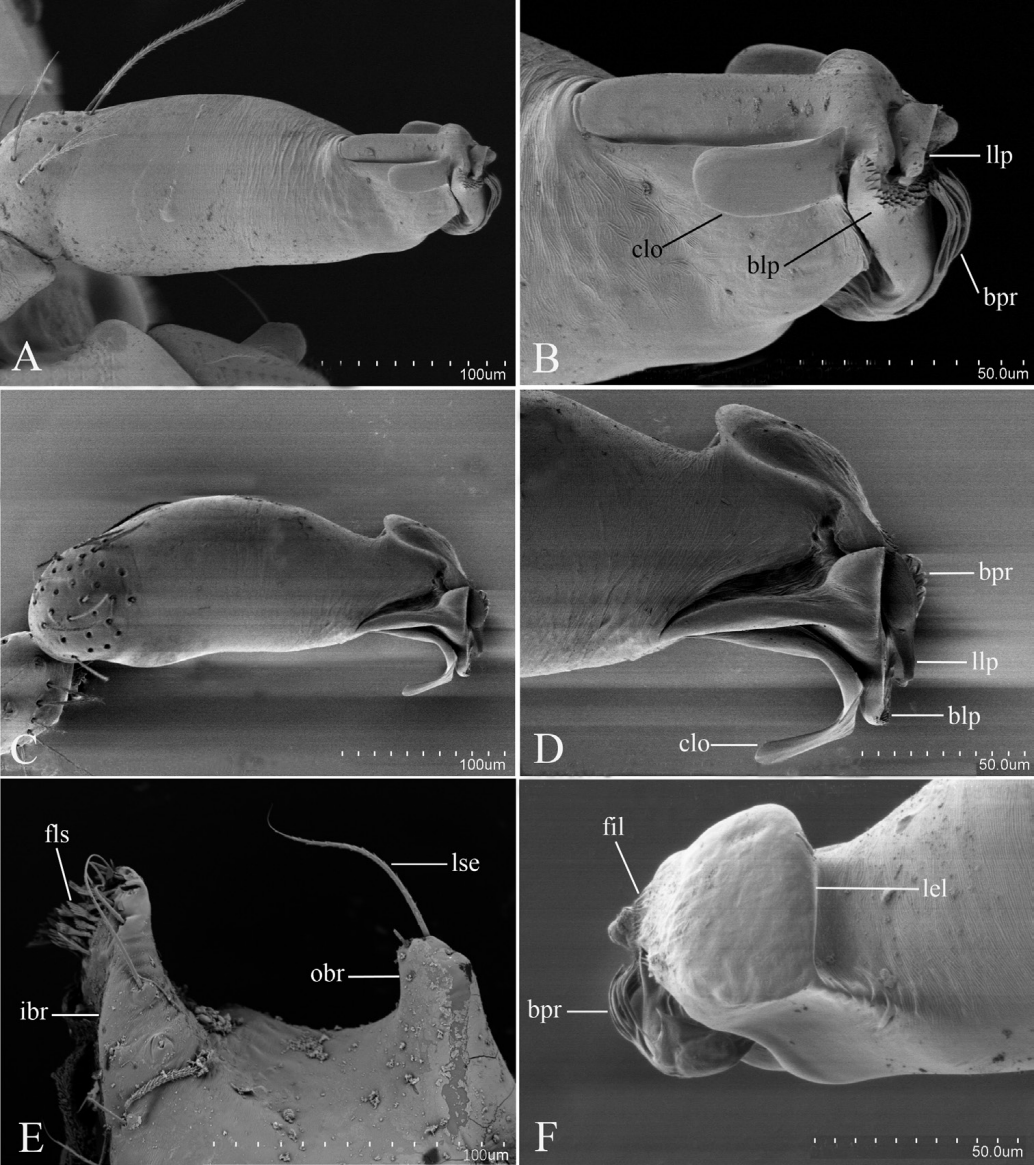
*Kachiniaputao* sp. n., male, SEM. **A, C** left palpl bulb, prolateral and dorsal views **B, D, F** embolus complex, prolateral, dorsal and retrolateral views **E** distal part of endite, ventral view. Abbreviations: blp = broom-like projection; bpr = brush-like projection; clo = curved lobe; fil = filiform lobe; fls = feather-like setae; ibr = inner branch; lel = large, ear-shaped lobe; llp = leaf-like projection; lse = long setae; obr = outer branch.

Male. Body yellow-brown, legs yellow. *Carapace* (Figs 1A, E, 4A, E): broadly oval in dorsal view, with brown egg-shaped patches behind eyes, eyes rather low; pars cephalica strongly elevated, pars thoracica higher than pars cephalica, with rounded posterolateral corners, posterolateral edge without pits, posterior margin not bulging below posterior rim, anterolateral corners without extension or projections, posterolateral surface without spikes, surface of pars cephalica smooth, thorax without depressions, fovea absent, without radiating rows of pits; lateral margin straight, smooth, rebordered, with small blunt denticles; marginal setae present. *Eyes* (Figs 1B, H, 4B, H): six, well developed, arranged in a compact group; ALE largest, PME, PLE subequal; ALE–PLE separated by less than ALE radius, PME touching each other; posterior row recurved from above, procurved from front. *Clypeus* (Figs 1F, H, 4F, H): margin unmodified, sinuous in front view, vertical in lateral view, median projection absent; light setae, needlelike. Chilum absent. *Mouthparts* (Figs 1G, 2E, 5E): chelicerae straight; labium rectangular, anterior margin deeply incised (ldi), same as sternum in sclerotization, not fused to sternum; endites slender, anterior margin with a row of small serrula, distally branched, with dense, patch of short feather-like setae (fls) on inner branch (ibr) and two long setae (lse) on outer branch (obr). *Sternum* (Figs 1C, D, 4C, D): longer than wide, with radial furrows between coxae, uniform, not fused to carapace, median concavity absent, surface smooth, anterior margin unmodified, posterior margin not extending posteriorly of coxae IV, anterior corner unmodified, distance between coxae approximately equal, lateral margins unmodified, without posterior hump; setae sparse, dark, needlelike, evenly scattered, without hair tufts. *Abdomen* (Figs 1C, E, 4C, E): ovoid, rounded posteriorly. Dorsal scutum covering entire dorsum, strongly sclerotized, without pattern. Epigastric scutum strongly sclerotized, surrounding pedicel. Postgastric scutum strongly sclerotized, long, almost rectangular, covering nearly the full length of the abdomen, anterior margin unmodified, with posteriorly directed lateral apodemes. Book lung covers large, smooth, anterolateral edge unmodified. Scutopedicel region has short tube, scutum not extending far beyond dorsum of pedicel, plumose hairs absent. Anterior spiracles connected (Figure 1C) or not (Figure 4G) by a furrow. Postgastric scutum with (Figure 4E, G) or without (Figure 1C, E) a cluster of strong, long setae (sls). Spinneret scutum without fringe of setae. *Legs* (Figs 1A, C, 4A, C): without pattern; patella plus tibia I longer than carapace. Leg spines: tibiae I, II with four pairs of ventral spines each; metatarsi I, II with two pairs of ventral spines each, legs III and IV without spines. *Genitalia* (Figs 1C, 4G): epigastric region with sperm pore small, oval, rebordered, situated between anterior and posterior spiracles. *Palp* (Figs 1I–K, 4I–K): strongly sclerotized, right and left palps symmetrical. Trochanter with a ventral protuberance (vp). Cymbium almost totally fused with bulb. Embolus complex (Figs 2B, D, F, 5B, D, F) complicated, distal part with several projections.

**Figure 3.**
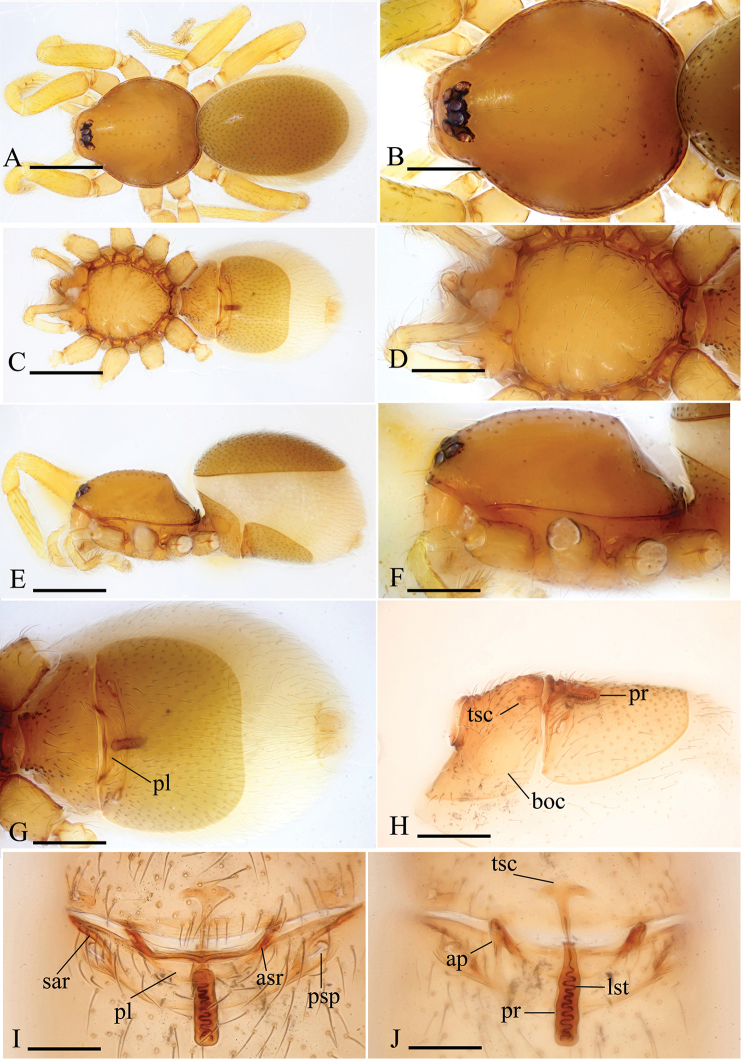
*Kachiniaputao* sp. n., paratype female. **A, C, E** habitus, dorsal, ventral and lateral views **B, D, F** prosoma, dorsal, ventral and lateral views **G, H** abdomen, ventral and lateral views **I, J** genitalia, ventral and dorsal views. Abbreviations: ap = apodemes; asr = anterior scutal ridge; boc = booklung cover; lst = line-like structure; pl = plate; pr = posterior receptacle; psp = posterior spiracles; sar = sclerotized, recurved arches; tsc = T-shaped sclerite. Scale bars: 0.4 mm (**A, C, E**); 0.2 mm (**B, D, F, G, H**); 0.1 mm (**I, J**).

Female. As in male except as noted. Dorsal and postgastric scuta smaller than in male. Palp without claw; spines absent. Labium and endites unmodified. *Abdomen* (Figs 3A, 6A): dorsal scutum large, covering more than 5/6 of dorsum. Postgastric scutum rectangular, not fused to epigastric scutum (Figs 3C, 6C). Posterior spiracles connected by groove; with two strongly sclerotized, recurved arches (sar) anterior to the posterior spiracles. *Genitalia*: surface without external features (Figs 3I, 6I). Dorsal view (Figs 3J, 6J) with a T-shaped sclerite (tsc) anteriorly, followed posteriorly by a tube-like posterior receptacle (pr); lateral apodemes (ap) present.

#### Composition.

*Kachiniaputao* Tong & Li, sp. n. and *K.mahmolae* Tong & Li, sp. n.

#### Distribution.

Myanmar (Kachin State).

### Kachinia putao

Taxon classificationAnimaliaAraneaeOonopidae

Tong & Li sp. n.

http://zoobank.org/CBE0BE63-B464-42F3-93B3-788345452D38

Figs 1 , 2 , 3

#### Type material.

**Holotype**: ♂ (IZCAS Ar-25090), Myanmar, Kachin State, Putao, around Ziradum Village, 27°33.465'N, 97°06.580'E, 1051 m, Wu J. & Chen Z., 8.V.2017. **Paratypes**: 5♂, 13♀ (IZCAS Ar-25091), same data as holotype; 2♀ (IZCAS Ar-25094), same data as holotype; 6♂, 6♀ (IZCAS Ar-25092), Myanmar, Kachin State, Putao, Hponkanrazi Wildlife Sanctuary, around Ziradum, 27°34.499'N, 97°05.546'E, 1106 m, Wu J., 19.X.2016; 6♂, 4♀ (IZCAS Ar-25094), same data as for holotype.

#### Etymology.

The species epithet, a noun in apposition, refers to the type locality.

#### Diagnosis.

The new species is similar to *K.mahmolae* sp. n., but can be distinguished by the proportion of the cymbiobulbus (length/maximal width = 3) (Figure 1I), the flat, wide and elongated lobe of the embolus complex (clo, Figure 2B, D) and the absence of long, strong setae on the abdominal scutum in males, and the presence of a crescent-shaped plate (pl, Figure 3G, I) of the endogyne.

#### Description.

Male (holotype). Habitus as in Figure 1A, C, E. Body length 1.59; carapace 0.75 long, 0.67 wide; abdomen 0.78 long, 0.51 wide.

Palp (Figure 1I–K): Femur 0.32 long, 0.17 width, length/maximal width = 1.88. Cymbiobulbus 0.57 long, 0.19 wide, length/maximal width = 3. Embolus complex (Figure 2B, D, F) with four projections from prolateral view, including a flat, wide and elongated, strongly curved lobe (clo), a leaf-like projection (llp), a broom-like projection (blp), and a long, brush-like projection (bpr); with a large, ear-shaped lobe (lel) in retrolaeral view, the distal end with filiform lobe (fil).

Female. Habitus as in Figure 3A, C, E. Body length 1.61; carapace 0.69 long, 0.62 wide; abdomen 0.94 long, 0.65 wide. Postgastric scutum 0.40 long, 0.46 width, length/width ratio = 0.87.

Genitalia. Ventral view (Figure 3I): middle part of anterior margin of postgastric scutum strongly sclerotized (asr), with a narrow, crescent-shaped plate (pl). Dorsal view (Figure 3J): with a T-shaped sclerite (tsc) anteriorly, followed posteriorly by a tube-like posterior receptacle (pr); from lateral view, the tube curved ventrally, then extends anteriorly, ending at the crescent-shaped plate, the ending point nearly reaching anterior margin of postgastric scutum (Figure 3G, H, I); a very thin, long and line-like structure (lst) can be seen inside the tube.

#### Distribution.

Myanmar (Kachin State).

### Kachinia mahmolae

Taxon classificationAnimaliaAraneaeOonopidae

Tong & Li sp. n.

http://zoobank.org/4229EC75-64B5-4066-8A2D-54334DC46503

Figs 4 , 5 , 6

#### Type material.

**Holotype**: ♂ (IZCAS Ar-25095), Myanmar, Kachin State, Putao, Mahmolae Village, 21°23.211'N, 97°21.485'E, 415 m, Wu J. & Chen Z., 5.V.2017. **Paratypes**: 1♂, 2♀ (IZCAS Ar-25096), same data as holotype; 1♂ (IZCAS Ar-25097), Myanmar, Kachin State, Putao, roadside between Nahteukhu and BaAve, 27°18.000'N, 97°23.267'E, 535 m, Wu J., 8.X.2016.

#### Etymology.

The species epithet, a noun in apposition, refers to the type locality.

#### Diagnosis.

**Figure 4.**
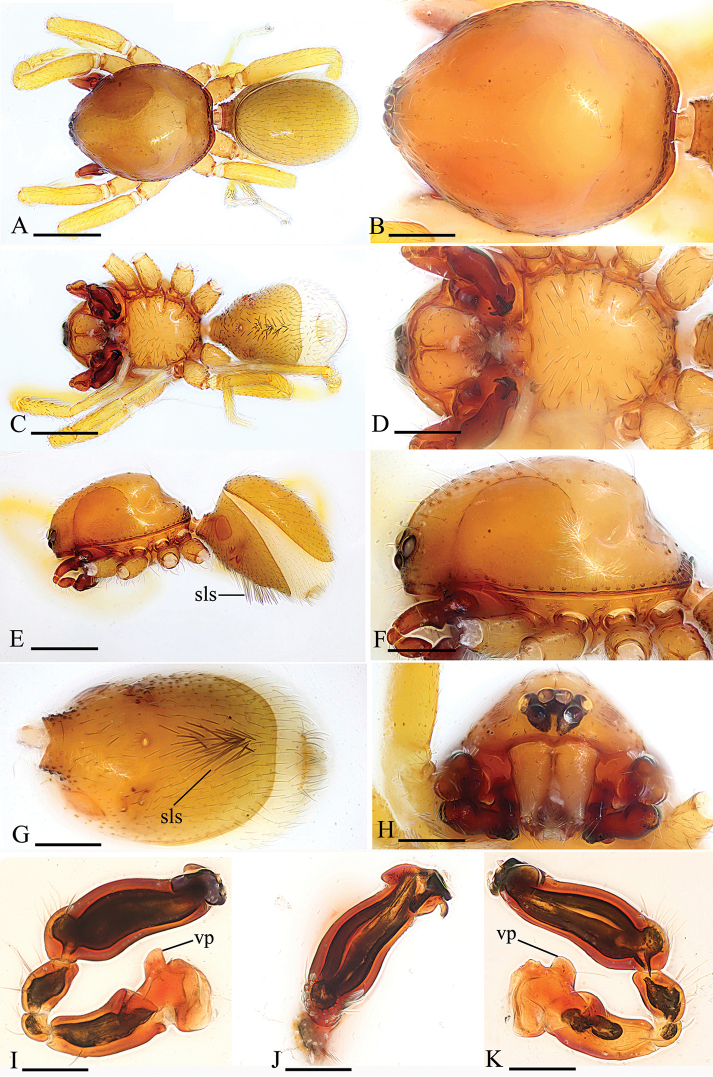
*Kachiniamahmolae* Tong & Li sp. n., holotype male. **A, C, E** habitus, dorsal, ventral and lateral views **B, D, F, H** prosoma, dorsal, ventral, lateral and anterior views **G** abdomen, ventral view **I, J, K** left palp, prolateral, dorsal and retrolateral views. Abbreviation: sls = strong, long setae; vp = ventral protuberance. Scale bars: 0.4 mm (**A, C, E**); 0.2 mm (**B, D, F, G, H**); 0.1 mm (**I, J, K**).

**Figure 5.**
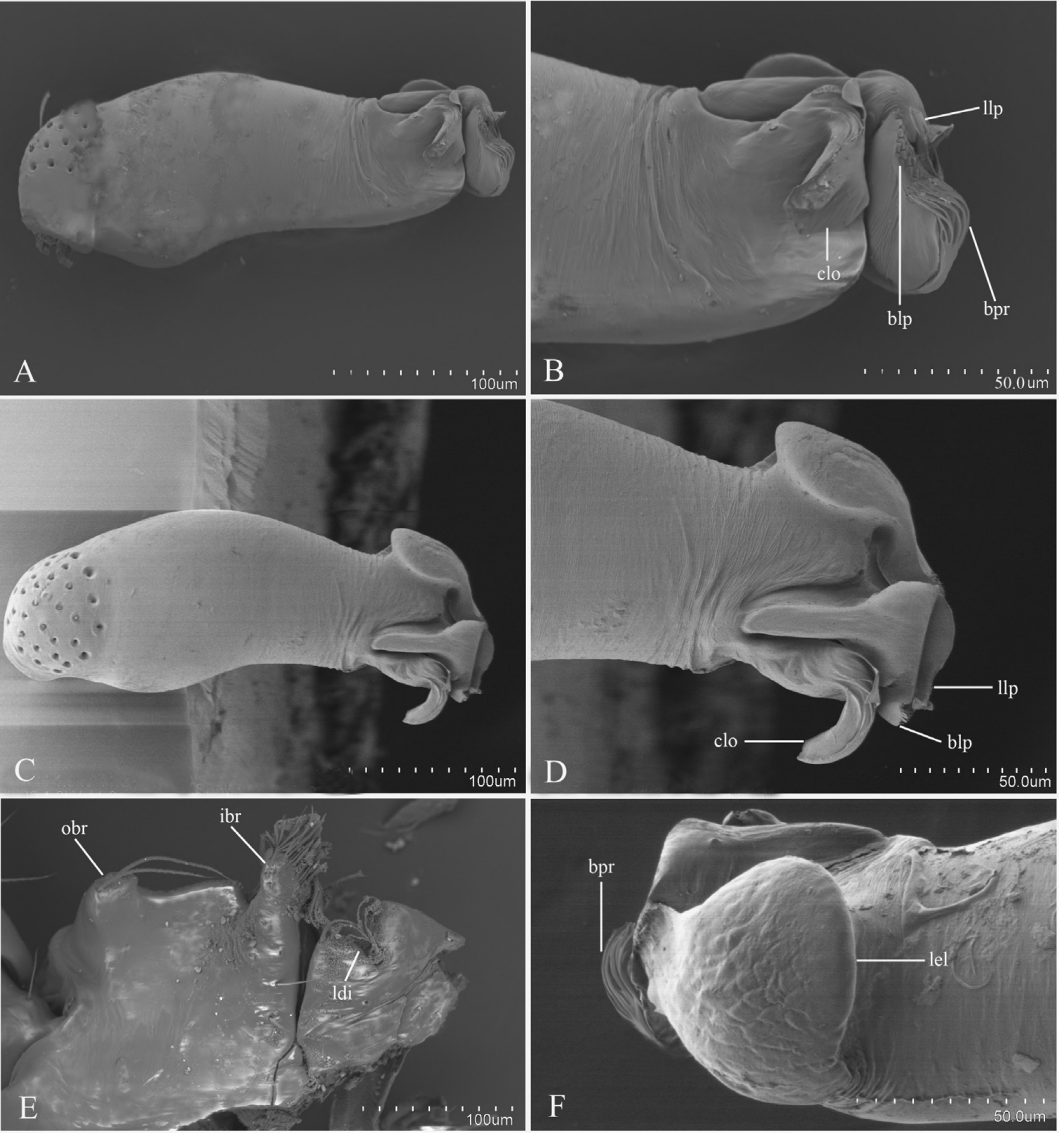
*Kachiniamahmolae* Tong & Li sp. n., male, SEM. **A, C** left palpal bulb, prolateral and dorsal views **B, D, F** embolus complex, prolateral, dorsal and retrolateral views **E** detached labium and endite, ventral view. Abbreviations: blp = broom-like projection; bpr = brush-like projection; clo = curved lobe; ibr = inner branch; ldi = labium deep incision; lel = large, ear-shaped lobe; llp = leaf-like projection; obr = outer branch.

**Figure 6.**
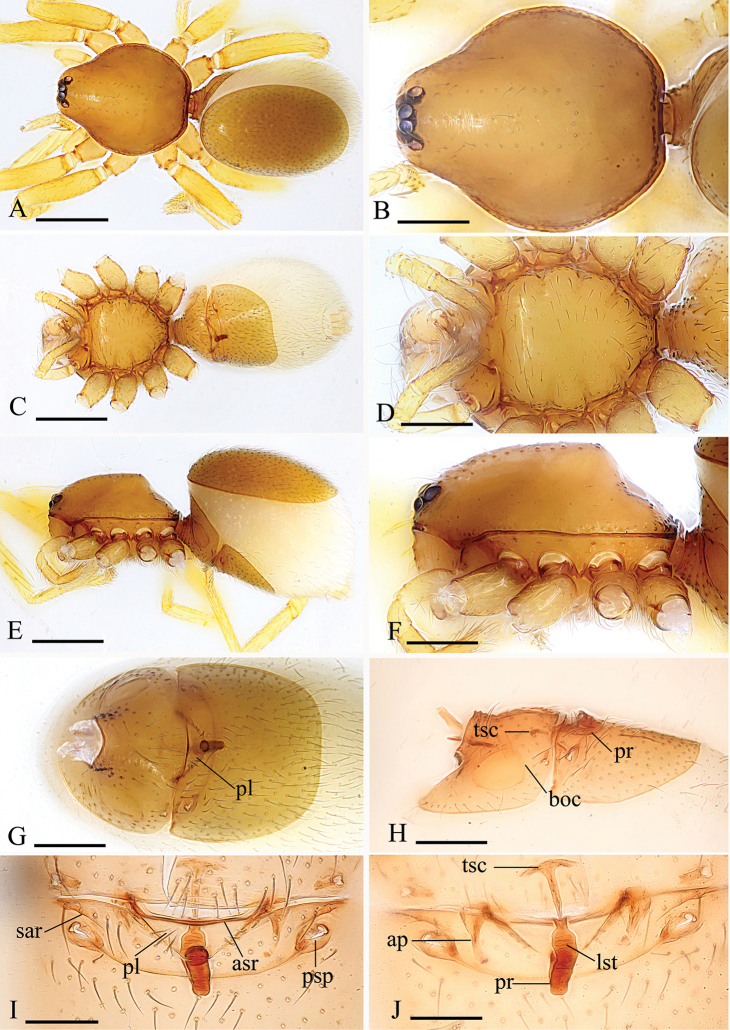
*Kachiniamahmolae* sp. n., paratype female. **A, C, E** habitus, dorsal, ventral and lateral views **B, D, F** prosoma, dorsal, ventral, and lateral views **G, H** abdomen, ventral and lateral views **I, J** genitalia, ventral and dorsal views. Abbreviations: ap = apodemes; asr = anterior scutal ridge; boc = booklung cover; lst = line-like structure; pl = plate; pr = posterior receptacle; psp = posterior spiracles; sar = sclerotized, recurved arches; tsc = T-shaped sclerite. Scale bars: 0.4 mm (**A, C, E**); 0.2 mm (**B, D, F, G, H**); 0.1 mm (**I, J**).

The new species is similar to *K.putao* sp. n. but can be distinguished by the proportion of the cymbiobulbus (length/maximal width = 2.6) (Figure 4I), the collapsed lobe of the embolus complex (clo, Figure 5B, D) and the long, strong setae on the abdominal scutum (sls, Figure 4E, G) in males, and the presence of a triangular plate (pl, Figure 6G, I) of the endogyne.

#### Description.

Male (holotype). Habitus as in Figure 4A, C, E. Body length 1.76; carapace 0.87 long, 0.72 wide; abdomen 0.78 long, 0.56 wide.

Palp (Figure 4I–K): Femur 0.28 long, 0.14 width, length/maximal width = 2. Cymbiobulbus 0.42 long, 0.16 wide, length/maximal width = 2.6. Embolus complex (Figure 5B, D, F) with four projections from prolateral view, including a collapsed, strongly curved lobe (clo), a leaf-like projection (llp), a broom-like projection (blp), and a long, brush-like projection (bpr); with a large, ear-shaped lobe (lel) in retrolateral view, the distal end with filiform lobe (fil).

Female. Habitus as in Figure 6A, C, E. Body length 1.72; carapace 0.75 long, 0.64 wide; abdomen 0.91 long, 0.67 wide. Postgastric scutum 0.34 long, 0.45 width, length/width ratio = 0.75.

Genitalia. Ventral view (Figure 6G, I): middle part of anterior margin of postgastric scutum strongly sclerotized (asr), with a triangular plate (pl). Dorsal view (Figure 6J): with a T-shaped sclerite (tsc) anteriorly, followed posteriorly by a tube-like posterior receptacle (pr) (Figure 6I); from lateral view, the receptacle curves ventrally, then extends anteriorly, ending at the triangular plate, the ending point far away the anterior margin of postgastric scutum (Figure 6G, H, I); a very thin, long and line-like structure (lst) can be seen inside the tube.

#### Distribution.

Myanmar (Kachin State).

## Supplementary Material

XML Treatment for Kachinia

XML Treatment for Kachinia putao

XML Treatment for Kachinia mahmolae
